# A case report of *CAT* gene and *HNF1β* gene variations in a patient with early-onset diabetes

**DOI:** 10.1515/biol-2022-0026

**Published:** 2022-04-06

**Authors:** Tao Cui, Hai-Bing Ju, Peng-Fei Liu, Yun-Jun Ma, Fu-Xian Zhang

**Affiliations:** Department of Endocrine, 920th Hospital of Joint Logistics Support Force of the Chinese People’s Libration Army, Xishan District, Kunming 650032, China; Department of Ophthalmology, Western Theater Command Air Force Hospital, Chengdu 610000, China; Department of Psychiatry, 920th Hospital of Joint Logistics Support Force of the Chinese People’s Libration Army, Kunming 650032, China

**Keywords:** diabetes, genetic mutations, catalase, hepatocyte nuclear factor, prognosis

## Abstract

Complex forms of diabetes are the ultimate common pathway involving multiple genetic variations and multiple environmental factors. Type 2 diabetes (T2DM) is classified as complex diabetes. Varying degrees of insulin deficiency and tissue insulin resistance are two key links to T2DM. The islet β cell dysfunction plays a crucial role in the pathogenesis of T2DM. The decompensation of the islet β cell to insulin resistance is a common mechanism leading to the pathogenesis of T2DM. Available data show that genetic factors mainly affect cell function. At present, a number of susceptibility genes related to T2DM have been reported at home and abroad. In this study, the diabetes-related genes in the case of early-onset diabetes with a significant family history were examined, and our results showed the presence of the intron mutations of catalase (*CAT*) gene and hepatocyte nuclear factor 1β (*HNF1β*) gene. The patient enrolled in this study was observed and analyzed, thus, increasing further understanding of the genes associated with diabetes and exploring the pathogenesis of diabetes from the molecular level. This is significant for guiding the prevention, treatment, and prognosis evaluation of diabetes.

## 1 Introduction

Diabetes mellitus has become a major disease, impacting greatly on the lives and health of patients. Moreover, diabetes is a common chronic disease in older patients. Worldwide, 425 million adults have diabetes, with the highest prevalence of diabetes among those aged 65–79 years [1]. Diabetes involves multiple genetic variants and environmental factors. These pathways involve insufficient insulin secretion or insulin resistance through pancreatic β-cell tissue. Type 2 diabetes (T2DM) is a complex form of diabetes and is reported to be related to some susceptible genes.

## 2 Case presentation

### 2.1 Introduction and medical history

The patient was a 28-year-old woman, an only child, delivered normally and spontaneously, and her parents’ marriage was nonconsanguineous. She was born with a weight of 3.5 kg, developed obesity in early childhood, and had normal intelligence. At the age of 18 years, the patient was admitted to a local hospital for “cellulitis of the right lower limb,” during which hyperglycemia was detected. Her fasting blood glucose was 10.5 mmol/L at the time, and she was diagnosed with “T2DM.” There was no diabetic ketoacidosis at the time of the diagnosis. All the antibodies of glutamic acid decarboxylase antibody (GADA), Islet cell antibody (ICA), and insulin autoantibody (IAA) were negative. In the early stage of the disease, she was treated with oral hypoglycemic drugs such as metformin and gliclazide irregularly, without strict lifestyle intervention, and her blood glucose control was poor, up to 32.9 mmol/L. At the age of 24 years, the patient repeatedly suffered from “diabetic ketoacidosis” and “diabetic ketosis” and began insulin treatment. The patient’s daily dose of insulin ranged from 40 to 50 units, and her blood glucose control was not ideal (16.0–20.0 mmol/L). Then, the patient developed peripheral neuropathy symptoms such as cooling, numbness, and needle-pricking pain. Meanwhile, clinical urine protein (5.0 g/24 h) was found in the patient at the age of 25 years. At this time, she was also diagnosed with diabetic kidney disease and dyslipidemia. One year later, there was a rise in her serum creatinine (108 µmol/L) and hypoalbuminemia with no evidence of autoimmune nephropathy or glomerulonephritis and normal levels of anti-nuclear antibody, anti-neutrophellol cytoplasmic antibody, Complement 3, Complement 4, rheumatoid factor, uric acid, C-reactive protein, and serum immunoglobulin. One year later, the patient had edema on both lower limbs and hypertension and began to take antihypertensive drugs (nifedipine controlled-release tablets, 30 mg oral qd).


**Informed consent:** Informed consent has been obtained from all individuals included in this study.
**Ethical approval:** The research related to human use has been complied with all the relevant national regulations, institutional policies, and in accordance with the tenets of the Helsinki Declaration, and has been approved by the authors’ institutional review board or equivalent committee.

### 2.2 Family history

**Figure 1:**
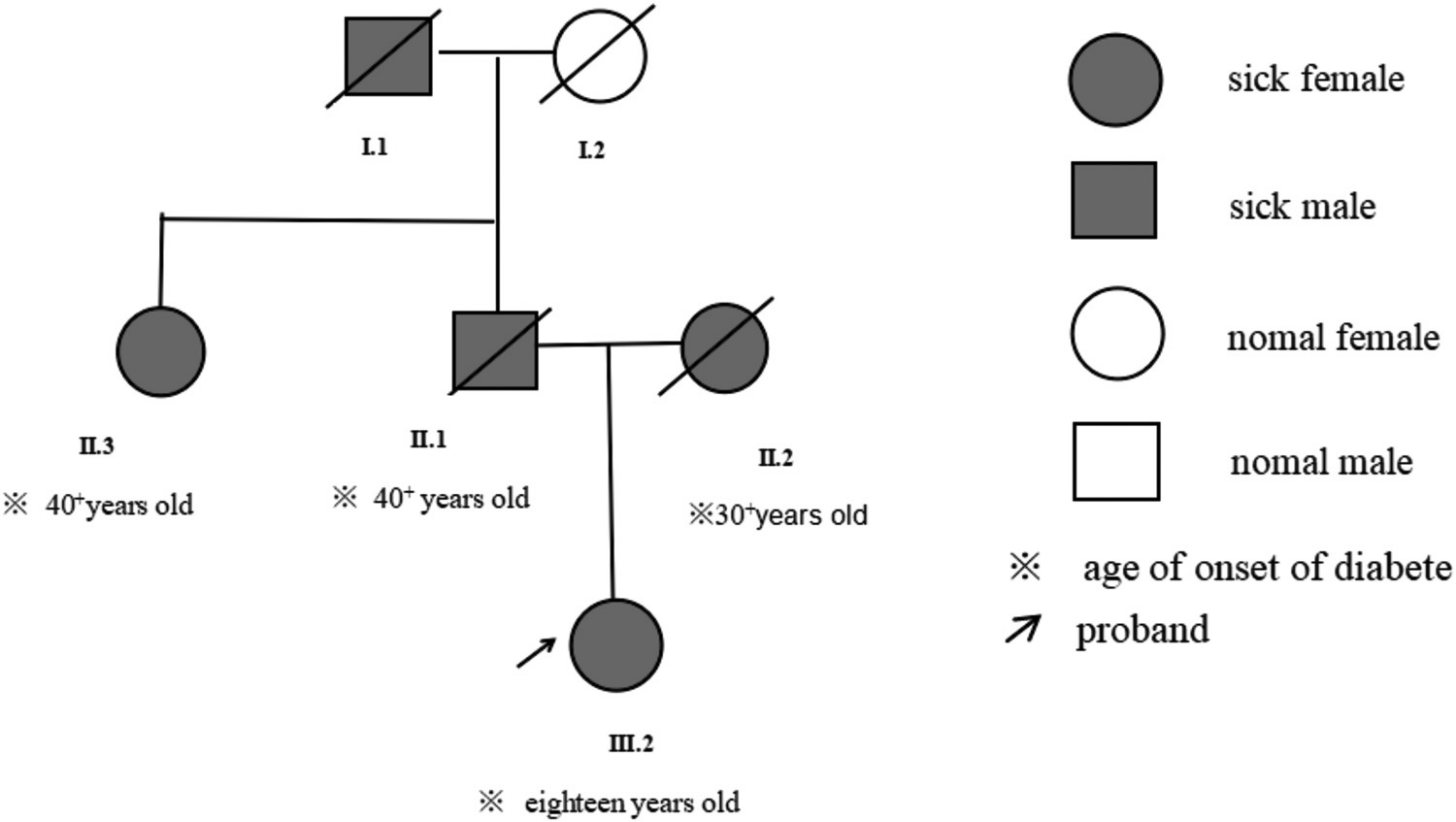
Genetic pedigree of the proband.

Her grandfather, father, mother, and aunt had diabetes (Figure 1). Her father was diagnosed with diabetes in his 40s and died of lung cancer. Her mother was diagnosed with diabetes in her 30s–40s and died of renal failure. Her aunt, father, and mother were all obese. In the early years of the disease, their clinical manifestations were not prominent, and they were treated with oral hypoglycemic drugs. However, their management of diabetes was lax, which may have contributed to the early onset of complications. Except for the deceased grandfather, whose medical history could not be traced, all were diagnosed with T2DM in the local hospital. Unfortunately, however, examination and test data for their diagnosis are not available.

### 2.3 Physical examination

The patient had a clear mind, normal intelligence, normal development, a height of 161 cm, a bodyweight of 85 kg, a body mass index of 32.79 kg/m^2^, uniformity of obesity, mild facial edema, no obvious positive signs in cardio, pulmonary, or abdominal examinations, symmetrical moderate edema in both lower limbs, and symmetrical pulsation of the dorsal artery in the feet.

### 2.4 Laboratory examination

The patient’s biochemical indicators were tested, and an insulin and C-peptide release test was performed to evaluate the level of C-peptide secretion. Multiple laboratory findings were abnormal. The patient’s fasting blood glucose was 11.7 mmol/L (normal reference values 3.9–6.0 mmol/L), glycosylated hemoglobin was 9.1% (4.0–6.0%), creatinine was 307 µmol/L (44–97 µmol/L), glomerular filtration rate was 22.67 mL/min (80–120 mL/min), serum cystatin C was 3.0 mg/L (0.5–1.1 mg/L), hemoglobin was 111 g/L (120–150 g/L), albumin was 22.2 g/L (35–55 g/L), triglyceride was 4.47 mmol/L (0.23–1.69 mmol/L), total cholesterol was 6.83 mmol/L (2.86–5.98 mmol/L), low-density lipoprotein cholesterol was 4.04 mmol/L (0.90–3.10 mmol/L), urine ketone body was positive, and 24 h urine protein was 10.37 g/24 h (<0.15 g/24 h). GADA, ICA, and IAA were all negative. The patient’s insulin and C-peptide release test showed that there was a certain level of C-peptide secretion, but the peak disappeared, indicating that the function of the islet β cell was relatively damaged (Table 1).

**Table 1:** The level of blood glucose, insulin and C-P (steamed bread meal test)

	Fasting	1 h	2 h
Blood glucose (mmol/L)	11.7	20.3	17.6
Insulin (uIU/mL)	10.10 (0.86–11.03)	46.63 (10.54–61.03)	40.22 (1.02–41.05)
C-peptide (ng/mL)	2.58 (0.52–4.38)	2.97 (3.58–13.27)	3.01 (1.20–11.36)

### 2.5 Imaging examination

Ultrasound: Fatty liver, no abnormality was found in either of the patient’s kidneys, her heart size was normal, her diastolic function decreased, and atherosclerosis was found in both lower extremities. Examination of the ocular fundus: diabetic retinopathy was found in both of the patient’s eyes (stage 3). The patient objected to renal biopsy, so the renal pathological results could not be obtained, and diabetic nephropathy could not be diagnosed definitely.

### 2.6 Diagnosis

1. Diabetes (T2DM, more likely); 1.1 Diabetic ketoses, 1.2 Diabetic peripheral neuropathy, 1.3 Diabetic kidney disease (stage V), and 1.4 Diabetic retinopathy (stage 3). 2. Hypertension level II (very high risk); 3. Dyslipidemia; 4. Hypoproteinemia.

### 2.7 Genetic detection methods

DNA was extracted from the patient’s peripheral blood by using the column method. Through sequencing technology, direct sequencing was performed on a variety of gene exon coding regions, which were compared with reference sequences to find possible gene mutations. The bioinformatics analysis method used included the analysis of missense mutations: PolyPhen2 (polymorphism type); sorting intolerant from tolerant, likelihood ratio test, mutation taster, mutation assessor, functional analysis through hidden Markov models; genomic evolutionary rate profiling; Phylop; and SiPhy and the analysis of splicing changes: NetGene2 Server, AUGUSTUS.

### 2.8 Genetic detection results

**Figure 2:**
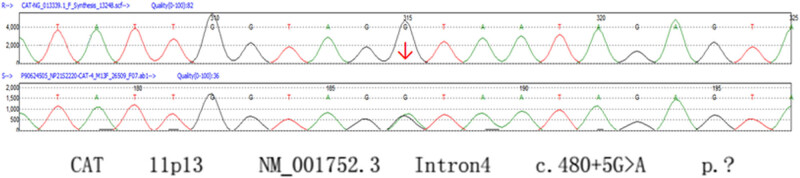
The results of gene detection (CAT).

**Figure 3:**
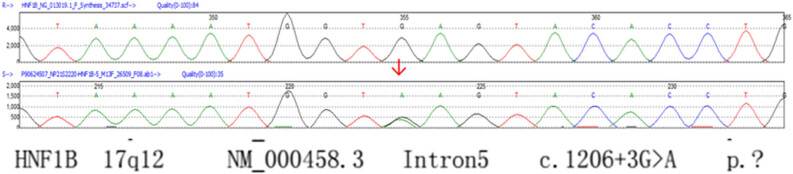
The results of gene detection (HNF-1β).

The detection results of the genes: a G to A substitution at the fifth position of intron 4 (a splicing mutation) was found in the catalase (*CAT*) gene, and a G to A substitution at the third position of intron 5 (a splicing mutation) was found in the *HNF1β* gene (Table 2 and Figures 2 and 3).

**Table 2:** The results of gene detection

Gene	Chromosomal location	Transcription	Position	cDNA level	Protein level	Variation
*CAT*	11p13	NM_001752.3	Intron4	c.480 + 5G > A p.	Heterozygosis	Suspicious of pathogenic
*HNF1B*	17q12	NM_000458.3	Intron5	c.1206 + 3G > A p.	Heterozygosis	Meaning is unknown

## 3 Discussion

### 3.1 The *CAT* gene and diabetes

CAT is one of the key enzymes in the biological antioxidation defense enzyme system. It can promote the decomposition of H_2_O_2_ into molecular oxygen and water and remove H_2_O_2_ from the body to protect the cells from the toxicity of H_2_O_2_. In addition to its recognized oxidant action, CAT can be used as a physiological signal transduction molecule [1]. CAT is expected to modulate the H_2_O_2_-dependent signal transduction pathway by affecting the cell level of hydrogen peroxide. To a certain extent, CAT content in tissues or plasma can reflect the state of oxidative stress and the antioxidant capacity of the organ. A growing number of studies have confirmed the correlation between oxidative stress and insulin resistance in obesity and T2DM [2,3,4,5,6]. Excessive free radical production or inadequate free radical scavenging for any reason can lead to the rise of free radicals, which, in turn, can lead to lipid peroxidation stress injury that is associated with the pathophysiological process of diabetes. Cellular antioxidant activity, including CAT, may be key to this process [7]. So far, there have been many studies on the correlation between single nucleotide polymorphisms (SNP) in the *CAT* gene and diseases, suggesting that genetic variations in CAT and its promoters are risk factors for a variety of cardiometabolic diseases [8], such as diabetes, hypertension, dyslipidemia, and arterial aging [9,10,11,12]. Decreased mRNA and protein expression of CAT may enhance reactive oxygen species-induced cell damage through the accumulation of H_2_O_2_ [13,14]. Several studies showed that the activity of CAT in patients with diabetes decreased significantly compared with healthy people and led to microvascular complications (e.g., diabetic retinopathy) in patients with diabetes [15,16,17,18,19].

In the present case, the *CAT* gene variations of the proband were splicing mutation. According to the preliminary analysis of cell function research and bioinformatics software, the mutation significantly affects mRNA splicing and is expected to cause changes in the splicing site, resulting in the disorder of the encoded protein and loss of its normal function. ESP6500siv2_ALL and 1000-thousand genome (1000g2015aug_ALL) database are not included, while the dbSNP147 database is included (rs761650208). In 1990, Wen et al. [20] detected the same mutation in a Japanese-type acatalasemia, and the same splicing site mutation was found in the genomic DNA of another individual from an unrelated family. The mutation was believed to be the most likely cause of Japanese-type acatalasemia. Acatalasemia is a rare genetic deficiency in humans that involves severe reductions in catalase activity and is usually an autosomal recessive inheritance. Acatalasemia in humans is considered an asymptomatic disease [21], and catalase-deficient mice are described as being phenotypically normal [22]. Nevertheless, more recent epidemiological studies show that human subjects with acatalasemia have an increased risk of developing numerous pathologies, including altered lipid and carbohydrate metabolism and T2DM [23,24,25].

Heit et al. [26] reported the CAT-knockout mice showed increased fasting insulin levels, abnormal glucose tolerance, and more enlarged islets of Langerhans. It was predicted that CAT deficiency would adversely affect lipid trafficking and glucose homeostasis by promoting the accumulation of hydrogen peroxide and thereby eliciting oxidant damage or amplified signal transduction pathways [26]. These results provide evidence for the *CAT* gene being involved in insulin resistance and the development of a pre-diabetic state.

The patient in this study and her parents were obese, and she suffered from peripheral neuropathy, retinopathy, renal injury, and hypertension. Her mother had early-onset diabetes complicated with severe renal injury and eventually died of renal failure. So, it is speculated that in addition to the impact of long-term hyperglycemia, increased oxidative stress and insulin resistance related to genetic variation might be an enabling factor in the development of diabetes and its complications. Altogether, we consider this variation as a suspicious pathogenic variation. Unfortunately, because the patient’s parents had died, it was not possible to test their genes.

### 3.2 The *HNF1β* gene and diabetes

HNF1β is a member of the transcription factor superfamily containing homologous domains, which plays an important role in tissue-specific regulation of gene expression in the liver, pancreas, kidney, intestines, reproductive tract, and other organs, and also participates in the embryonic development of these organs. Heterozygous mutations of the *HNF1β* gene can lead to abnormalities of these organs, manifesting as various renal and extrarenal phenotypes. Their clinical manifestations can be isolated or multisystem involvement, and the severity of clinical phenotypes varies greatly among affected patients in the same family [27,28,29].

It is also expressed in the pancreatic cells, and its main target genes are pyruvate kinase, glucose transporter glucose transport-2, and the insulin gene [30]. The *HNF1β* gene plays an important role in the primary pathophysiology of T2DM. It was involved in the loss of neurogenin-3 (Ngn3)-positive endocrine progenitor cells, pancreatic atrophy, and reduced insulin sensitivity to endogenous glucose production leading to the reduction of insulin secretion [31]. Previous studies have shown that *HNF1β* gene mutations may be involved in late-onset common T2DM in addition to maturity-onset diabetes in young patients 5 [32]. Some genome-wide association studies revealed that several tag SNPs in the *HNF1β* gene were associated with the susceptibility of T2DM and such associations were well replicated in many countries [33,34,35]. Rs4430796 (A > G) in intron 2 of HNF1β is the most frequent SNP in the Chinese population [36]. Huang et al. first reported that the rs11651052-A allele increased the risk of T2DM by 1.26-fold compared with the G allele. The study also provided evidence that age, gender, BMI, smoking, and drinking status have an interactive effect with these variants on T2DM susceptibility [37]. The research described HNF1β-related diabetes and associated phenotypes and assessed genotype/phenotype correlations at diagnosis and in the long term. The study showed that in a cohort of 201 adult patients with HNF1β defects, diabetes was present in 159 patients. At follow-up, 79% of patients were treated with insulin, 44% had chronic kidney disease stage 3–4 (CKD3–4), 21% had end-stage renal disease (ESRD), and 122 of 166 patients had renal cysts. By multivariate analysis, CKD3-4/ESRD at follow-up was associated with HNF1β mutation. Diabetes complications, cardiovascular risk factors, chronic CKD3-4, and ESRD are highly prevalent in patients with HNF1β syndrome [38]. Some researchers analyzed the genotype–phenotype correlations in 14 pediatric patients with HNF1β mutations. Genetic studies revealed that all patients had bilateral renal abnormalities, primarily multiple renal cysts. Twelve patients exhibited progressive renal functional deterioration, and diabetes developed in five patients (36%) [39].

The gene variation in the present case was splicing mutation, which is expected to cause changes in the splicing sites and result in the disorder of the encoded protein and loss of its normal function. However, no literature report was found on the HGMD database, and no data were included in ESP6500siv2_ALL, dbSNP147, and 1000g2015aug_ALL. The preliminary analysis by bioinformatics software showed that the variation did not affect mRNA splicing, but the predicted results were for reference only. Although this variation has not been reported in the literature, in view of the correlation between the *HNF1β* gene and diabetes and renal impairment, it cannot be ruled out that it may be an important factor in promoting early-onset diabetes and nephropathy. We examined the patient’s ultrasound but found no renal cysts. However, her kidney function is significantly impaired. In general, it is not clear that this variation is a pathogenic gene, and its clinical significance needs to be studied further. At diabetes diagnosis, the presence of morphological or functional kidney disease may help etiological diagnosis. Genotype/phenotype correlations may have implications for the care and prognosis of these patients.

## 4 Conclusion

At present, the incidence of diabetes is occurring at a younger age in patients, the proportion of early-onset diabetes is higher, the incidence of diabetic microvascular disease is higher, and familial aggregation is more common. Therefore, the detection and screening of diabetes-related genes in patients and relatives with a significant family history of diabetes and the exploration of the pathogenesis of diabetes from the molecular level have significance in guiding the prevention, treatment, and prognosis evaluation of such diseases. After genetic testing and comprehensive analysis were completed, we speculated that the *CAT* gene variation was likely to be a pathogenic gene and the significance of the *HNF1β* gene variation was debatable. From the perspective of mechanism and relevant evidence, the *HNF1β* gene variation is highly likely to be associated with the occurrence and development of T2DM and promote renal failure. This needs to be demonstrated by more studies in the future.

## References

[1] Groeger G, Quiney C, Cotter TG. Hydrogen peroxide as a cell-survival signaling molecule. Antioxid Redox Signal. 2009;11:2655–71. 10.1089/ars.2009.2728 19558209

[2] Haldar SR, Chakrabarty A, Chowdhury S, Haldar A, Sengupta S, Bhattacharyya M. Oxidative stress-related genes in type 2 diabetes: association analysis and their clinical impact. Biochem Genet. 2015;53:93–119. 10.1007/s10528-015-9675-z 25991559

[3] Evans JL, Maddux BA, Goldfine ID. The molecular basis for oxidative stress-induced insulin resistance. Antioxid Redox Signal. 2005;7:1040–52. 10.1089/ars.2005.7.1040 15998259

[4] Furukawa S, Fujita T, Shimabukuro M, Iwaki M, Yamada Y, Nakajima Y, et al. Increased oxidative stress in obesity and its impact on metabolic syndrome. J Clin Invest. 2004;114:1752–61. 10.1172/JCI21625 PMC535065 15599400

[5] Ikemura M, Nishikawa M, Hyoudou K, Kobayashi Y, Yamashita F, Hashida M. Improvement of insulin resistance by removal of systemic hydrogen peroxide by PEGylated catalase in obese mice. Mol Pharm. 2010;7:2069–76. 10.1021/mp100110c 21033698

[6] Reuter S, Gupta SC, Chaturvedi MM, Aggarwal BB. Oxidative stress, inflammation, and cancer: how are they linked? Free Radic Biol Med. 2010;49:1603–16. 10.1016/j.freeradbiomed.2010.09.006 PMC2990475 20840865

[7] Rochette L, Lorin J, Zeller M, Guilland JC, Lorgis L, Cottin Y, et al. Nitric oxide synthase inhibition and oxidative stress in cardiovascular diseases: possible therapeutic targets? Pharmacol Ther. 2013;140:239–57. 10.1016/j.pharmthera.2013.07.004 23859953

[8] Zhou XF, Cui J, DeStefano AL, Chazaro I, Farrer LA, Manolis AJ, et al. Polymorphisms in the promoter region of catalase gene and essential hypertension. Dis Markers. 2005;21:3–7. 10.1155/2005/487014 PMC3851957 15735318

[9] Chen H, Yu M, Li M, Zhao R, Zhu Q, Zhou W, et al. Polymorphic variations in manganese superoxide dismutase (MnSOD), glutathione peroxidase-1 (GPX1), and catalase (CAT) contribute to elevated plasma triglyceride levels in Chinese patients with type 2 diabetes or diabetic cardiovascular disease. Mol Cell Biochem. 2012;363:85–91. 10.1007/s11010-011-1160-3 22167619

[10] Nivet-Antoine V, Labat C, El Shamieh S, Dulcire X, Cottart CH, Beaudeux JL, et al. Relationship between catalase haplotype and arterial aging. Atherosclerosis. 2013;227:100–5. 10.1016/j.atherosclerosis.2012.12.015 23340375

[11] Kasznicki J, Sliwinska A, Kosmalski M, Merecz A, Majsterek I, Drzewoski J. Genetic polymorphisms (Pro197Leu of Gpx1, +35A/C of SOD1, -262C/T of CAT), the level of antioxidant proteins (GPx1, SOD1, CAT) and the risk of distal symmetric polyneuropathy in Polish patients with type 2 diabetes mellitus. Adv Med Sci. 2016;61:123–9. 10.1016/j.advms.2015.10.006 26674569

[12] Cavarape A, Feletto F, Mercuri F, Quagliaro L, Daman G, Ceriello A. High-fructose diet decreases catalase mRNA levels in rat tissues. J Endocrinol Invest. 2001;24:838–45. 10.1007/BF03343940 11817707

[13] Maithili Karpaga Selvi N, Sridhar MG, Swaminathan RP, Sripradha R. Curcumin attenuates oxidative stress and activation of redox-sensitive kinases in high fructose- and high-fat-fed male wistar rats. Sci Pharm. 2014;83:159–75. 10.3797/scipharm.1408-16 PMC4727822 26839808

[14] Francini F, Castro MC, Schinella G, García ME, Maiztegui B, Raschia MA, et al. Changes induced by a fructose-rich diet on hepatic metabolism and the antioxidant system. Life Sci. 2010;86:965–71. 10.1016/j.lfs.2010.05.005 20470786

[15] Ghattas MH, Abo-Elmatty DM. Association of polymorphic markers of the catalase and superoxide dismutase genes with type 2 diabetes mellitus. DNA Cell Biol. 2012;31:1598–603. 10.1089/dna.2012.1739 22970972

[16] Gupta MM, Chari S. Lipid peroxidation and antioxidant status in patients with diabetic retinopathy. Indian J Physiol Pharmacol. 2005;49:187–92. 16170987

[17] Chen BH, Jiang DY, Tang LS. Advanced glycation end-products induce apoptosis involving the signaling pathways of oxidative stress in bovine retinal pericytes. Life Sci. 2006;79:1040–8. 10.1016/j.lfs.2006.03.020 16674981

[18] Chang D, Pan HZ, Xu FJ, Feng GL, Li H, Kuang HY, et al. Change of antioxidative enzymes and products of oxidative stress in diabetes mellitus and diabetic retinopathy. J Harbin Med Univ. 2008;42:475–8 [in Chinese].

[19] Hebert-Schuster M, Fabre EE, Nivet-Antoine V. Catalase polymorphisms and metabolic diseases. Curr Opin Clin Nutr Metab Care. 2012;15:397–402. 10.1097/MCO.0b013e328354a326 22617568

[20] Wen JK, Osumi T, Hashimoto T, Ogata M. Molecular analysis of human acatalasemia. Identification of a splicing mutation. J Mol Biol. 1990;211:383–93. 10.1016/0022-2836(90)90359-T 2308162

[21] Takahara S, Ogata M. Erythrocyte metabolism against oxidation in Japanese acatalasemia. Monogr Hum Genet. 1978;10:205–11. 10.1159/000401594 723895

[22] Ho YS, Xiong Y, Ma W, Spector A, Ho DS. Mice lacking catalase develop normally but show differential sensitivity to oxidant tissue injury. J Biol Chem. 2004;279:32804–12. 10.1074/jbc.M404800200 15178682

[23] Góth L, Rass P, Páy A. Catalase enzyme mutations and their association with diseases. Mol Diagn. 2004;8:141–9. 10.1007/BF03260057 15771551

[24] Góth L, Nagy T. Inherited catalase deficiency: is it benign or a factor in various age related disorders? Mutat Res. 2013;753:147–54. 10.1016/j.mrrev.2013.08.002 24025477

[25] Hwang I, Lee J, Huh JY, Park J, Lee HB, Ho YS, et al. Catalase deficiency accelerates diabetic renal injury through peroxisomal dysfunction. Diabetes. 2012;61:728–38. 10.2337/db11-0584 PMC3282807 22315314

[26] Heit C, Marshall S, Singh S, Yu X, Charkoftaki G, Zhao H, et al. Catalase deletion promotes prediabetic phenotype in mice. Free Radic Biol Med. 2017;103:48–56. 10.1016/j.freeradbiomed.2016.12.011 PMC5513671 27939935

[27] Clissold RL, Hamilton AJ, Hattersley AT, Ellard S, Bingham C. HNF1B-associated renal and extra-renal disease-an expanding clinical spectrum. Nat Rev Nephrol. 2015;11:102–12. 10.1038/nrneph.2014.232 25536396

[28] Bockenhauer D, Jaureguiberry G. HNF1B-associated clinical phenotypes: the kidney and beyond. Pediatr Nephrol. 2016;31:707–14. 10.1007/s00467-015-3142-2 26160100

[29] Verhave JC, Bech AP, Wetzels JF, Nijenhuis T. Hepatocyte nuclear factor 1β-associated kidney disease: more than renal cysts and diabetes. J Am Soc Nephrol. 2016;27:345–53. 10.1681/ASN.2015050544 PMC4731131 26319241

[30] Hani EH, Stoffers DA, Chèvre JC, Durand E, Stanojevic V, Dina C, et al. Defective mutations in the insulin promoter factor-1 (IPF-1) gene in late-onset type 2 diabetes mellitus. J Clin Invest. 1999;104:R41–8. 10.1172/JCI7469 PMC409821 10545531

[31] El-Khairi R, Vallier L. The role of hepatocyte nuclear factor 1β in disease and development. Diabetes Obes Metab. 2016;18(Suppl 1):23–32. 10.1111/dom.12715 27615128

[32] Dong Y, Luo M. Advances in molecular mechanism and clinical research of MODY. Int J Endocrinol Metab. 2003;23:240–2 [article in Chinese].

[33] Hara K, Shojima N, Hosoe J, Kadowaki T. Genetic architecture of type 2 diabetes. Biochem Biophys Res Commun. 2014;452:213–20. 10.1016/j.bbrc.2014.08.012 25111817

[34] DIAbetes Genetics Replication And Meta-analysis (DIAGRAM) Consortium, Asian Genetic Epidemiology Network Type 2 Diabetes (AGEN-T2D) Consortium, South Asian Type 2 Diabetes (SAT2D) Consortium, Mexican American Type 2 Diabetes (MAT2D) Consortium, Type 2 Diabetes Genetic Exploration by Nex-generation sequencing in muylti-Ethnic Samples (T2D-GENES) Consortium, Mahajan A, Go MJ, Zhang W, Below JE, Gaulton KJ, Ferreira T, et al. Genome-wide trans-ancestry meta-analysis provides insight into the genetic architecture of type 2 diabetes susceptibility. Nat Genet. 2014;46:234–44. 10.1038/ng.2897 PMC3969612 24509480

[35] Machado-Silva W, Tonet-Furioso AC, Gomes L, Córdova C, Moraes CF, Nóbrega OT. The rs4430796 SNP of the HNF1β gene associates with type 2 diabetes in older adults. Rev Assoc Med Bras. 1992;2018(64):586–9. 10.1590/1806-9282.64.07.586 30365659

[36] Li H, Gan W, Lu L, Dong X, Han X, Hu C, et al. A genome-wide association study identifies GRK5 and RASGRP1 as type 2 diabetes loci in Chinese Hans. Diabetes. 2013;62:291–8. 10.2337/db12-0454 PMC3526061 22961080

[37] Huang T, Wang L, Bai M, Zheng J, Yuan D, He Y, et al. Influence of IGF2BP2, HMG20A, and HNF1B genetic polymorphisms on the susceptibility to Type 2 diabetes mellitus in Chinese Han population. Biosci Rep. 2020;40:BSR20193955. 10.1042/BSR20193955 PMC7256674 32329795

[38] Dubois-Laforgue D, Cornu E, Saint-Martin C, Coste J, Bellanné-Chantelot C, Timsit J, et al. Diabetes, associated clinical spectrum, long-term prognosis, and genotype/phenotype correlations in 201 adult patients with hepatocyte nuclear factor 1B (HNF1B) molecular defects. Diabetes Care. 2017;40:1436–43. 10.2337/dc16-2462 28420700

[39] Lim SH, Kim JH, Han KH, Ahn YH, Kang HG, Ha IS, et al. Genotype and phenotype analyses in pediatric patients with HNF1B mutations. J Clin Med. 2020;9:2320. 10.3390/jcm9072320 PMC7408390 32708349

